# Incentive motivation improves numerosity discrimination in children and adolescents

**DOI:** 10.1038/s41598-022-14198-7

**Published:** 2022-06-16

**Authors:** Luca Spliethoff, Shu-Chen Li, Annika Dix

**Affiliations:** 1 Faculty of Psychology, Chair of Lifespan Developmental Neuroscience, Technische Universität Dresden, Zellescher Weg 17, 01062 Dresden, Germany; 2 Faculty of Education, Chair of Vocational Education, Technische Universität Dresden, Weberplatz 5, 01217 Dresden, Germany; 3 Centre for Tactile Internet with Human-in-the-Loop (CeTI), Technische Universität Dresden, 01062 Dresden, Germany; 4 Present Address: Faculty of Psychology, Chair of Engineering Psychology and Applied Cognitive Research, Technische Universität Dresden, Zellescher Weg 17, 01062 Dresden, Germany

**Keywords:** Cognitive neuroscience, Motivation, Reward, Sensory processing

## Abstract

We recently showed that incentive motivation improves the precision of the Approximate Number System (ANS) in young adults. To shed light on the development of incentive motivation, the present study investigated whether this effect and its underlying mechanisms may also be observed in younger samples. Specifically, seven-year-old children (*n* = 23; 12 girls) and 14-year-old adolescents (*n* = 30; 15 girls) performed a dot comparison task with monetary reward incentives. Both age groups showed higher accuracy in a reward compared to a neutral condition and, similarly, higher processing efficiency as revealed by the drift rate parameter of the EZ-diffusion model. Furthermore, in line with the Incentive Salience Hypothesis, phasic pupil dilations—indicating the activation of the brain’s salience network—were greater in incentivized trials in both age groups. Together these finding suggest that incentive modulation improves numerosity discrimination in children and adolescents by enhancing the perceptual saliency of numerosity information. However, the observed reward anticipation effects were less pronounced in children relative to adolescents. Furthermore, unlike previous findings regarding young adults, the decision thresholds of children and adolescents were not raised by the monetary reward, which may indicate a more protracted development of incentive regulation of response caution than perceptual evidence accumulation.

## Introduction

Representing and processing non-symbolic information of quantities are basic abilities for making sense of the environment^1,2^. Such cross-species abilities^3,4^ are ascribed to a language-independent cognitive system known as the Approximate Number System (ANS) that is present as early as 6 months of age in humans. The acuity of the ANS improves gradually during childhood and adolescence into adulthood^5–7^. Yet, even in adults, the ANS is characterized by imprecision^8^. To date, studies aiming at tuning the precision of the ANS are still scarce. Whereas in most of these studies performance was modulated via providing accuracy-based feedback^9,10^, we recently showed that young adults’ performance in a non-symbolic numerosity discrimination task could be enhanced by monetary reward incentives^11^. In order to shed light on the development of processes underlying motivational regulation of numerosity perception, the present study examined whether the precision of the ANS in younger participants, namely children and adolescents, could also be enhanced by rewards.

According to the Incentive Salience Hypothesis^12^, incentives enhance the perceptual salience of stimuli via reward-related mesolimbic dopamine (DA) signals, thereby modulating the sensory representation of the stimuli. Previous research has shown that neurobiological mechanisms including mesolimbic DA signalling as well as its relation to cognition and incentive motivation are subject to developmental effects^13–15^. Both childhood and adolescence are characterized by still maturing DA pathways, with insufficient mesolimbic and mesocortical DA signalling during childhood and an imbalance of signalling between these pathways during adolescence^13,16^. Given such developmental effects, it is of interest to investigate potential developmental differences in motivational regulation of numerosity perception.

Evidence from past studies about the effects of incentive motivation on cognition during childhood and adolescence indicates reward-enhancing effects in different domains. For instance, in an antisaccade task, performance-contingent monetary rewards improved inhibitory control in children and adolescents^17^. Similarly, another study revealed that reward contingencies significantly improved response inhibition accuracy in a sample of 8- to 12-year-old boys^18^. The observed effect was especially pronounced for monetary compared to social rewards, thus refuting the assumption that children are not yet sensitive to monetary rewards. Other effects of reward had also been observed in the arithmetic domain, where performance-contingent monetary rewards increased task performance in 8- to 11-year-old children^19^. For adolescents, reward-related improvements in response inhibition were shown by Geier, et al.^20^. Further, Hardin, et al.^21^ compared the effects of incentives on cognitive control in adults and adolescents and observed a greater impact of reward in adolescence than in adulthood, albeit incentives yielded positive effects in both age groups. However, to date, no studies have investigated potential developmental differences in incentive modulation of numerosity perception.

Individual and age-related differences in numerosity perception are commonly conceptualized as differences in the precision of the ANS that can be measured by non-symbolic numerical discrimination tasks^5,6^. In the dot comparison task, participants have to indicate which of the two presented dot clouds contains more dots. Task difficulty is varied by changing the ratio between the quantities of the dot clouds, where a lower ratio means a higher difficulty^11,22^ (discriminating dot clouds of 10 vs. 9 dots is more difficult than discriminating dot clouds of 4 vs. 3 dots). In the present study, we used a modified dot comparison task with trial-by-trial reward incentives^11^ to measure the precision of the ANS and its modulation by reward. Specifically, by applying a variant of the drift diffusion model (DDM), one of the model parameter, the drift rate (v), served as a measure of ANS precision^23^. The DDM is helpful in isolating the decision process into several distinct components and has gained increasing attention in the field of perceptual decision making and numeracy during the last years^24^. The model assumes decision making to be a noisy process during which perceptual information is gradually accumulated over time toward one of two response alternatives. The speed with which a decision can be reached depends on several factors, such as the saliency of the perceptual representation as well as an individual’s decision threshold. In developmental research, by taking potential developmental differences in speed-accuracy tradeoffs into account^25–27^, past studies applying the DDM have revealed specific age-related differences in perceptual decision making that go beyond the well-known lower accuracy and longer decision times of younger participants^28–30^.

Regarding numerosity perception, in particular, Park and Starns^23^ used a dot comparison task similar to ours and could show that the drift rate parameter of the DDM, an index of the efficiency of evidence-accumulation or quality of the extracted information, reliably captures the precision of the ANS and depends less on speed-accuracy tradeoff than the traditionally used Weber fraction^31^. Specifically, it was observed that the Weber fraction but not the drift rate was correlated with another parameter of the DDM, known as the decision threshold or boundary separation (a). This parameter reflects the amount of evidence that is necessary for a decision to be made for one of the options. Adapting the boundary separation (e.g. a more conservative response criterion) strongly influences response times (e.g. slower decision speed) and, thus, the speed-accuracy tradeoff in a task. Park and Starns^23^ showed that a wider boundary separation, which means an individual uses a more conservative decision criterion and takes more time for a decision, was also associated with a smaller Weber fraction spuriously suggesting higher ANS precision, which diminishes the validity of the Weber fraction compared to the drift rate parameter. Other than drift rate and decision boundary, a third parameter of the DDM known as non-decision time parameter (t_ER_) is thought to reflect all non-decision related processes, including stimulus encoding and response execution. Notably, in the context of numerosity discrimination, DDM-based analyses have indicated that children extract information lower in quality (i.e. less efficient evidence accumulation), while they also have a more conservative response style and spend more time on aspects like stimulus encoding or response execution compared to college students and adults^32^. Another study by Manning, et al.^26^ had also observed lower drift rates and wider boundary separations for younger children compared to older children and adults. In light of these previous findings, in the present study, we applied the DDM to investigate developmental differences in the effects of incentive motivation on numerosity discrimination. By doing so, we could examine developmental differences of incentive effects on more specific aspects of numerosity discrimination besides performance accuracy, response time, and basic perceptual threshold.

To shed light on the mechanisms underlying incentive motivation, we further assessed task-related pupil dilation (PD) in the present study, which has been associated with brain activity in the salience network during reward anticipation^33^. In line with the Adaptive Gain Theory (AGT) of locus coeruleus-norepinephrine (LC-NE) function^34^, research by Gilzenrat et al.^35^ indicates that pupillary activity reflects changes in the LC-NE system. More generally, pupil diameter seems rather the result of a complex interaction of different neurotransmitter systems, including dopaminergic functions^36^. Evidence for a specific link between reward and pupil diameter mediated via mesolimbic dopamine has been provided by a study on the influence of monetary reward on PD in patients with idiopathic Parkinson's syndrome^37^. In particular, it was shown that dopaminergic medication restored the patients' pupillary reward sensitivity in a speeded saccade task, suggesting that PD can be used as an indirect marker for dopamine-mediated autonomic reward effects. A recent study also showed that pupillometry reflects ageing-related differences in reward value sensitivity in a healthy ageing sample^38^. Moreover, studies with clinical samples provide evidence for pupillary reward sensitivity in younger participants^39,40^. For instance, DiCriscio and Troiani^39^ underscored that pupil measures are linked to individual differences in reward sensitivity in a 5- to 14-year-old sample. Of particular interest here is the recent evidence from Castaldi et al.^41^ suggesting that the pupil spontaneously responds to perceived numerosity. The participants in this study passively observed dots of different physical or illusory (i.e. perceived) numerosity—a grouping-based illusion was used in half of the dot arrays resulting in an underestimation of perceived numerosity. Variations in pupil size in response to the arrays were modulated by changes in perceived numerosity. Given this recent finding, besides its more well-known feature in reflecting effects of incentive motivation, pupil dilation is also sensitive to numerosity perception. Thus, pupil size appears to be a suitable psychophysiological measure for the effects of reward and age on numerosity perception.

**Figure 1:**
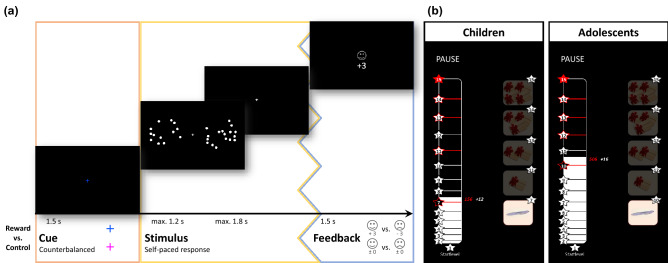
Incentivized dot comparison task: (**a**) Trial scheme showing the three phases (i.e. incentive cue, stimulus, feedback) of a correctly answered trial for the reward condition and below to it corresponding information on the control condition; (**b**) Scoring scheme shown to children (left) and adolescents (right) after each block—points collected in reward trials during the last block (white number), the accumulated total points (red number and white bar) as well as the reward level (level 1–15) with the corresponding win option (right panel).

Taken together, the present study seeks to examine the effects of incentive motivation on numerosity discrimination in children and adolescents as well as age-related differences in potential modulatory effects of reward. Independent of incentive-related performance modulation, we expected a main effect of age, with children showing worse numerosity perception both in terms of performance accuracy and discrimination speed in a dot comparison task (see Fig. 1 and the Methods section for more details). Other than these raw behavioural measures, in order to further investigate potential age-related differences, reward effects and their potential interactions, we applied a simplified version of the DDM, the EZ-diffusion model^42^, to decompose perceptual discrimination into three subprocesses. In light of previous findings reviewed above, we anticipated lower drift rates, wider boundary separations, and slower non-decision times for children than for adolescents. Beyond age effects, for both children and adolescents, we also expected reward-related improvements in performance, which would be indicated by higher discrimination accuracy as well as increased drift rates reflecting enhanced precision of the ANS. As for the age by incentive interaction, given lifespan development of dopamine function^13,43^ and previous research^44^ showing developmental differences in reward modulation of visual attention, we expected the effects of incentive to be larger in adolescents than in children. Other than measures of behavioural performance, effects of age and incentive modulation were also investigated with respect to pupil size.

## Results

### Behavioural performance

**Figure 2:**
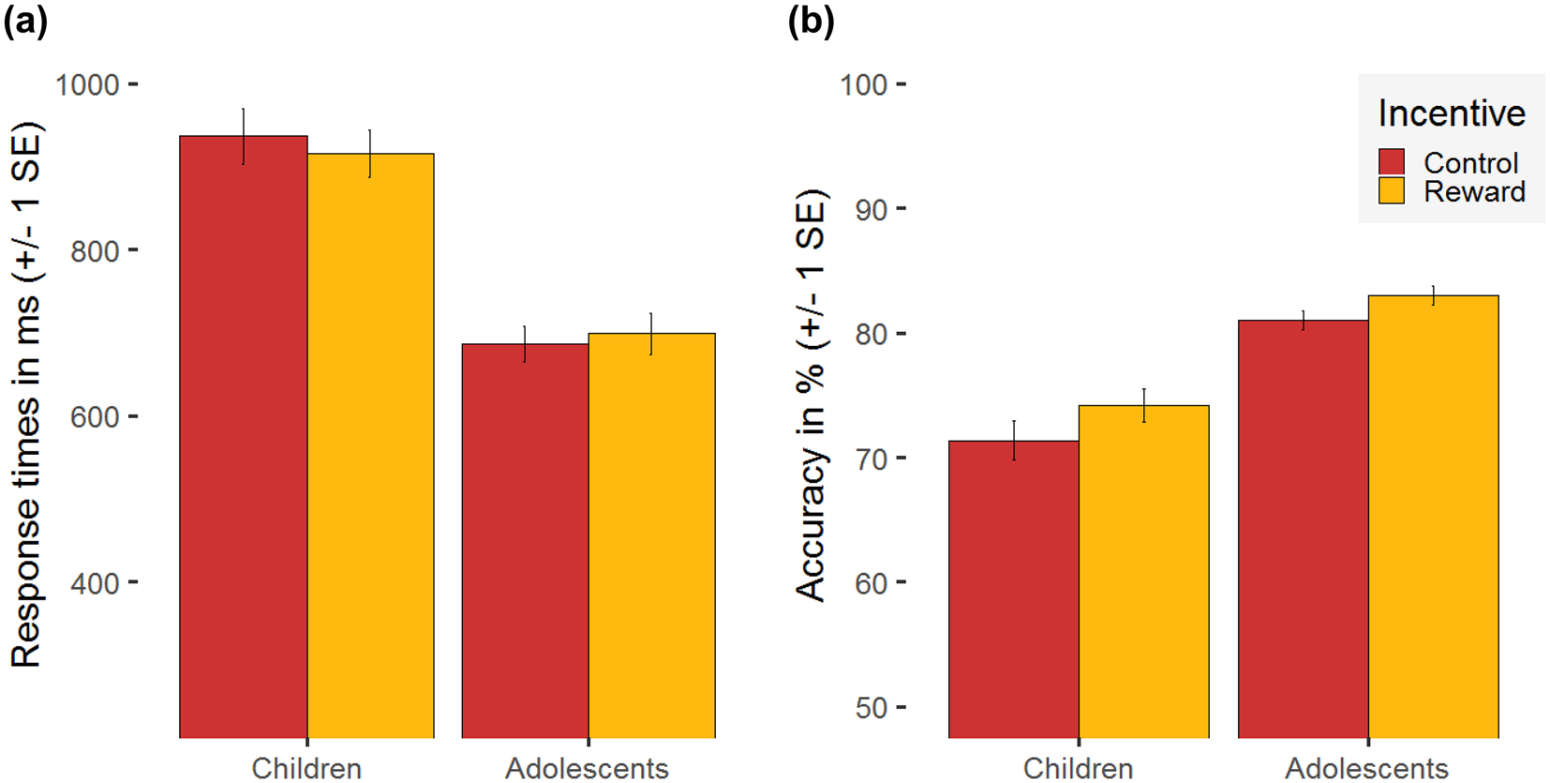
Behavioural performance: Mean and standard error (SE) for **(a**) response times (RT) in milliseconds and (**b**) accuracy in percent, both separated for the two incentive conditions (reward vs. control) and the two age groups (children vs. adolescents).

The linear mixed effects model with RTs as the dependent variable revealed a main effect of Age Group, *F*(1,51) = 39.00, *p* < 0.0001, η_p_^2^ = 0.43. While the main effect of Incentive did not reach significance (*p* = 0.71), the Incentive × Age Group interaction effect was significant, *F*(1,51) = 9.35, *p* < 0.01, η_p_^2^ = 0.15. Adolescents decided faster than children in both reward conditions (reward: *t*(47.90) = − 5.69, *p* < 0.0001; control: *t*(38.64) = − 6.42, *p* < 0.0001). Whereas reward marginally slowed the decision time in adolescents (*t*(29) = 1.87, *p* = 0.07, two-tailed, or *p* = 0.04, one-tailed), numerosity discrimination in children was faster in the reward compared to the control condition (*t*(29) = − 2.33, *p* = 0.03; see Fig. 2a). The linear mixed effects model for accuracy revealed main effects of Incentive, *F*(1,51) = 16.40, *p* < 0.001, η_p_^2^ = 0.24, and Age Group, *F*(1,51) = 42.30, *p* < 0.0001, η_p_^2^ = 0.45. The interaction Incentive × Age Group was not significant (*p* = 0.52). Adolescents answered more correctly than children and both groups showed better performance in trials of the reward compared to the control condition (see Fig. 2b).

### Model parameters of the decision-making process

**Figure 3:**
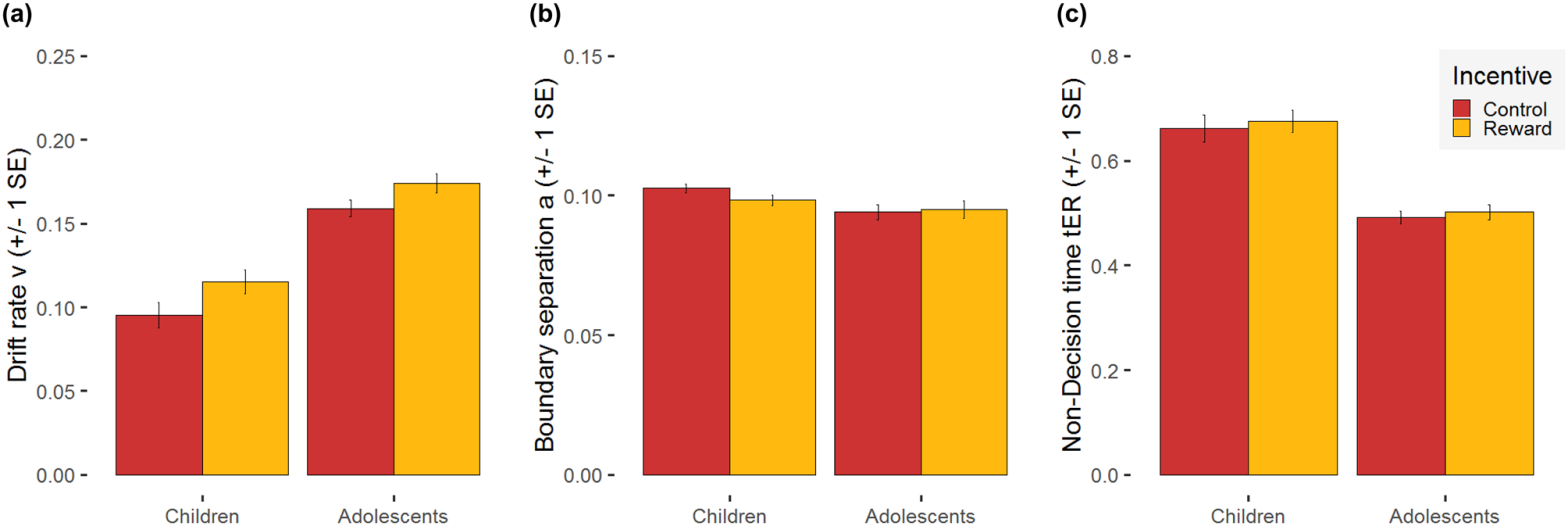
Model parameters of the decision-making process: Mean and standard error (SE) for (**a**) drift rate (v), (**b**) boundary separation (**a**), and (**c**) non-decision time (t_ER_), separated for the two incentive conditions (reward vs. control) and the two age groups (children vs. adolescents).

The linear mixed effects model for drift rate (v) of the DDM revealed main effects of Incentive, *F*(1,51) = 24.58, *p* < 0.001, η_p_^2^ = 0.33, and Age Group, *F*(1,51) = 57.86, *p* < 0.0001, η_p_^2^ = 0.53. The interaction Incentive × Age Group was not significant (*p* = 0.51). Adolescents’ information integration rate was higher than that of children. Further, in both groups, the drift rate was higher in the reward condition compared to the control condition (see Fig. 3a). The linear mixed effects model for boundary separation (a) only revealed a significant Incentive × Age Group interaction, *F*(1,51) = 9.63, *p* < 0.01, η_p_^2^ = 0.16, while main effects of Incentive and Age Group failed to reach significance (both *p*s = 0.11). The response criterion was less conservative (i.e. smaller boundary separation) for adolescents compared to children, but only in the control condition (*t*(43.24) = − 2.68, *p* = 0.03). In the reward condition, age groups did not differ in their response criterion (*p* = 0.36) as children lowered the criterion (i.e. became less conservative) in trials with reward compared to the control condition (*t*(43.24) = − 2.68, *p* = 0.03), whereas adolescents did not show any reduction (*p* = 0.41; see Fig. 3b). The linear mixed effects model for non-decision time (t_ER_) revealed main effects of Incentive, *F*(1,51) = 5.65, *p* = 0.02, η_p_^2^ = 0.10, and Age Group, *F*(1,51) = 45.57, *p* < 0.0001, η_p_^2^ = 0.47. The interaction Incentive × Age Group was not significant (*p* = 0.72). The non-decision time was longer (i.e. larger value of t_ER_) for children compared to adolescents and in trials with reward compared to control trials (see Fig. 3c).

### Pupillometry measures

**Figure 4:**
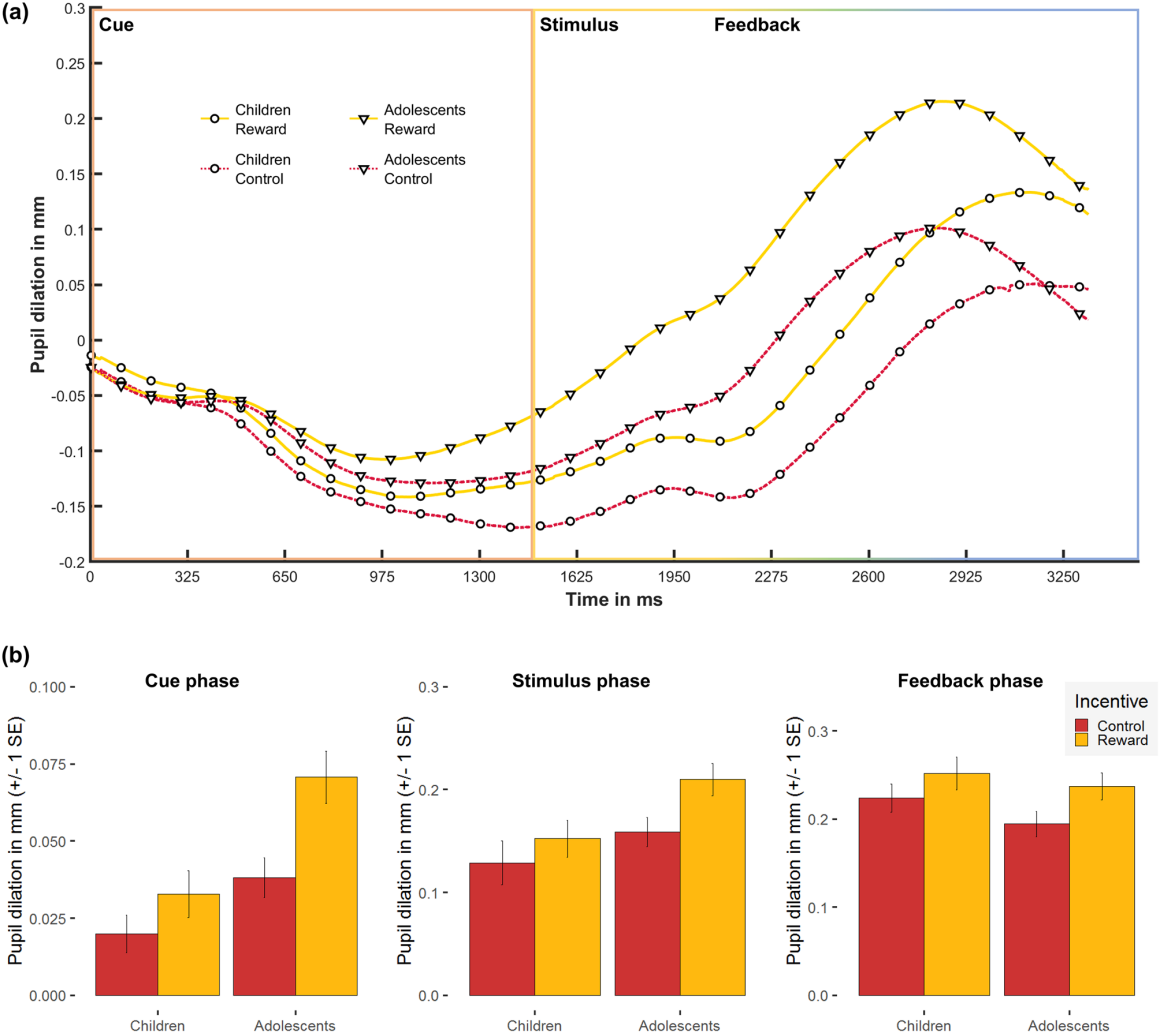
Pupillometry measures: (**a**) Mean pupil response (pupil dilation in millimetre) across the time course of the trial (in milliseconds) and the associated phase, stimulus-locked to the onset of the cue (zero point in time) and separated for the two incentive conditions (reward vs. control) and age groups (children vs. adolescents); (**b**) mean and standard error (SE) for the pupil dilation (in millimetres) measured at the peak of the pupil response during reward anticipation (cue phase; left), numerosity discrimination (stimulus phase; middle), and processing of the outcome (feedback phase; right), separated for the two incentive conditions (reward vs. control) and age groups (children vs. adolescents).

The average time courses of the pupil response over a trial for the different conditions are shown in Fig. 4a. The linear mixed effects models for peak PD revealed main effects of Incentive during reward anticipation (cue phase), *F*(1,51) = 31.72, *p* < 0.0001, η_p_^2^ = 0.38, numerosity discrimination (stimulus phase), *F*(1,51) = 29.00, *p* < 0.0001, η_p_^2^ = 0.36, and processing of the outcome (feedback phase), *F*(1,51) = 22.01, *p* < 0.0001, η_p_^2^ = 0.30. The PD was larger for trials with reward compared to the control condition in all three phases of the task (see Fig. 4b). Further, during the cue phase, the main effect of Age Group, *F*(1,51) = 8.61, *p* < 0.0001, η_p_^2^ = 0.14, as well as the interaction Incentive × Age Group, *F*(1,51) = 5.20, *p* < 0.0001, η_p_^2^ = 0.09, were significant. The PD was larger in adolescents than in children (reward: *t*(50.97) = 3.34, *p* < 0.01; control: *t*(50.71) = 2.06, *p* < 0.05). Moreover, in adolescents the pupil diameter increased more for trials with reward compared to the control condition, *t*(29) = 5.70, *p* < 0.0001). However, in children, the effect of Incentive during the cue phase just failed to reach significance (*p* = 0.06). In the two later task phases (stimulus and feedback phases), both the main effect of Age Group and the interaction Incentive × Age Group did not reach significance (stimulus phase: *p*s = 0.07; feedback phase: *p*s = 0.30–0.35). For further details regarding descriptive statistics of all dependent variables, see Table S5 in Supplementary Results.

## Discussion

In the present study, we investigated the effects of incentive motivation on numerosity discrimination in children and adolescents. Participants performed an incentivized non-symbolic dot comparison task with performance-contingent monetary rewards. Besides assessing both performance accuracy and reaction times, we also decomposed the decision processes into three distinct sub-processes by applying the EZ-diffusion model to the performance data. To shed light on psychophysiological mechanisms underlying potential modulatory reward effects, we also assessed phasic pupil dilations. We expected reward incentives to enhance the perceptual salience of the stimuli, thereby improving performance in the numerosity discrimination task in both age groups.

The main findings of the study are as follows: In both age groups, reward incentives improved discrimination accuracy and increased the drift rate indicating a positive effect of reward on the precision of the ANS. Reward modulations were also apparent at the psychophysiological level. Both children and adolescents showed larger pupil dilations in rewarded than in control trials in all three trial phases (cue, stimulus, feedback). Regarding the effect of age, as expected children performed poorer than adolescents independent of incentives: their responses were slower and less accurate. This age-related difference was also reflected in the parameters of the EZ-diffusion model. Children showed smaller drift rates and longer non-decision times. Although the two age groups did not differ in their response caution in general, an Age Group × Incentive interaction was observed. Children showed slower and more conservative responses (i.e. larger boundary separation) compared to adolescents in control trials. In rewarded trials, the value of the boundary separation parameter (i.e. decision threshold) was reduced in children and did not differ from that of adolescents. Although the effects of incentive motivation on pupil dilation were observed in both groups, a stronger reward-related modulation of the pupil size during the cue phase was found in adolescents compared to children, with children showing only a marginal effect of incentive. These results are largely in line with our hypotheses, which we discuss in more detail in what follows.

Earlier research has shown reward incentives to enhance behavioural performance in different cognitive and sensory domains^45–47^. More recently, Dix and Li^11^ could extend these findings to the domain of numerosity perception, by demonstrating increased discrimination accuracy and improved ANS precision (i.e. steeper drift rates) in adults in an incentivized numerosity discrimination task. Using the same task, we could replicate this effect within the present study for a group of 7-year-old children and 14-year-old adolescents. In both age groups, incentivized trials were associated with an increase in accuracy and steeper drift rates. In line with the Incentive Salience Hypothesis^12^, our results suggest that reward cues at the beginning of the trials enhance the perceptual salience of upcoming stimuli, thereby facilitating the discrimination of the two dot clouds. The reward incentives seem to influence the early stages of visual processing of the dot clouds before the quantities are represented in the cross-modality ANS.

This interpretation is also supported by results from the pupillary data of the present study. In trials where the cue indicated that a correct answer would involve a reward, pupil dilations were greater, compared to trials, in which cues indicated a neutral condition without reward. As shown by Schneider et al.^33^, pupil dilations that are linked to the anticipation of a monetary reward, are associated with increased activity in the dorsal anterior cingulate cortex (dACC). Forming part of the brain’s salience network, the dACC is involved in the bottom-up detection of salient information^48^. As claimed by the AGT^34^, the ACC further has the role of allocating attentional resources and, via direct projections to the LC, monitoring and optimizing task performance^49^. Activity of the LC, in turn, is directly linked to changes in pupil diameter^35^. This mechanism may be one explanation for participants’ increased ANS precision (i.e. steeper drift rates) in incentivized trials. Moreover, our results support the notion that pupil diameter is sensitive not only to LC-NE activity. Based on the finding of Manohar and Husain^37^, who demonstrated that pupil reward sensitivity is mediated via mesolimbic dopamine, the reward effect on pupil diameter in the present study suggests the involvement of dopaminergic processes. This is also consistent with previous findings suggesting that younger participants’ pupil diameter serves as an indicator of autonomic reward effects^39,40^. Overall, whereas findings from Castaldi et al.^41^ highlighted the spontaneous sensitivity of the pupil to perceived numerosity, we were able to extend their findings by demonstrating that the pupillary response during numerosity perception is also sensitive to reward modulations.

Admittedly, the reward effect on ANS precision could be also mediated through the enhancement of other cognitive processes with inhibitory control leading the way—we already mentioned findings on improved inhibitory control under reward^17^. Of note, inhibitory control has been also linked with the performance in non-symbolic numerical discrimination tasks due to the similarities with a Stroop task: the visual characteristics of the dot clouds such as dot size or area as well as sparsity can interfere with the numerical information^50^. However, evidence on this is not univocal: only a few studies directly report correlations between inhibitory control and ANS precision and results on this relationship are mixed^51,52^. Further, rather than being a mediator, we assume that inhibitory control abilities are similarly modulated by reward as ANS precision is, namely in the early stages of stimulus evaluation. For instance, Krebs et al.^53^ showed that monetary incentives associated with task-relevant features (colour) enhance performance in a Stroop task whereas monetary incentives associated with task-irrelevant features (semantics) impede task performance. The authors suggest that dopaminergic pathways may increase the salience of the relevant stimulus property facilitating its processing and thereby reducing the conflict. Accordingly, even if inhibitory control plays a critical role in the present study, the reward-induced performance improvements suggest that incentive salience can also facilitate the processing of numerical stimulus features to the detriment of other task-irrelevant visual features.

Our finding that children were slower and less accurate than adolescents in discriminating the non-symbolic quantities, substantiates past research suggesting that ANS acuity improves gradually until adulthood^5–7^. Fitting the EZ-diffusion model to our data allowed us to get a more detailed picture of reward effects and age-related differences concerning the distinct decision sub-processes, as it considers both accuracy and response time data. In line with prior research^26,32^, adolescents’ steeper drift rates indicate that they were more efficient than children in processing and extracting numerical information of high quality. Further, as expected and in line with prior research, children spent more time (i.e. longer t_ER_) on decision-irrelevant sensorimotor processes like stimulus encoding or response preparation^32^. Since the EZ-diffusion model decomposes the perceptual discrimination process and thereby accounts for these age differences in basic perceptual speed and motor preparation, we argue that the modulation of the drift rate by incentives suggests similar reward effects on ANS precision in both age groups. However, it has to be noted that the non-decision time is an underspecified term and little research exists so far about its specific meaning^54^.

Regarding the incentive regulation of the response caution, modulations of the decision threshold have been associated with the efficacy of nucleus caudate-cortex connections^55^. These connections undergo maturation until adulthood and are consequently not yet fully developed during adolescence^56^. This might explain why we did not find a significant increase in the boundary separation in incentivized trials in our sample, other than in young adults^11^—and relatedly, only a trend towards slower responses under reward, which was only apparent in adolescents but not in children. Future studies combining behavioural data on numerosity perception with structural and functional brain measures could help to substantiate this interpretation. For children, we found even an opposite effect on response caution with reward. In line with research on developmental differences in response styles^32^, in the neutral condition, children differ from adolescents by showing a more conservative decision criterion (i.e. wider boundary separation). Yet, in incentivized trials, children adapt their boundary separation by reducing it, indicating that under reward they tend to rely on less discriminative information before deciding which of the two dot clouds was larger. In contrast to the drift rate—a parameter that reflects factors affecting the efficiency of the evidence accumulation strategy—the boundary separation designates response style or strategy. Consequently, the instructions given a priori to a task can affect the boundary separation^57^. In the present study, participants were told to respond as quickly and accurately as possible. The results’ pattern indicates that children—unlike adolescents—might have generally adapted their response style in incentivized trials, by responding not only through enhancing accuracy but also by improving response speed, even though this was not the strategy leading to the highest monetary win. This might be due to the fact that the concept of money is only fully developed at age eight^58,59^ and thus children’s monetary awareness might differ from that of the adolescent sample. Considering the pupillary data could help to explain this effect further. During the cue phase, children’s pupil dilations were less modulated by reward incentives compared to adolescents, which underlines the less pronounced reward anticipation effects in these participants.

This finding is also in line with a large body of research indicating that substantial changes in the mesolimbic dopamine system occur during the period of adolescence, resulting in increased activity in reward-related brain regions^60,61^. Following the argumentation of Telzer^61^, the pronounced PD in reaction to the cue might challenge the deficit-oriented perspective of adolescents’ heightened reward sensitivity, as their discrimination performance improved with reward incentives. However, not only adolescents but also the group of children profited from rewards in terms of more accurate responses and better information accumulation. This also implies that the heightened pupillary reward effect in adolescents in the reward anticipation phase is not reflected in the behavioural performance, for which other processes than reward anticipation and corresponding adjustments in response strategy might play a critical role as well. In Dix and Li^11^, we suggest two processes that may underlie reward-related modulation of numerosity discrimination: (i) the LC-NE driven gain modulation of stimulus processing due to incentive salience; and (ii) a strategic process via top-down control by the prefrontal cortex (cf. adaptive regulation of performance as proposed by the AGT^34^). The findings of the present study regarding age-dependent modulations of the boundary separation parameter and the PD during reward anticipation (i.e. in the cue phase) suggest that only the second more domain-general process might be subject to developmental changes. In contrast, incentive salience seems to facilitate the processing of numerical stimulus features, as reflected in steeper drift rates for rewarded trials, irrespective of participants’ age. Besides these developmental insights into different mechanisms underlying effects of incentive motivation on numerosity discrimination, our results also indicate that both of the proposed underlying processes are reflected in the pupil response but with varying strengths at different processing phases. It can be speculated that, other than in the cue phase, pupil dilations during numerosity processing (i.e. in the stimulus and probably, at times, in the feedback phase) mainly reflect early perceptual processing of numerical information and their modulation by incentive motivation. Castaldi, et al.^41^ showed that the gain of the pupillary response spontaneously reflects a greater salience for stimuli of higher (illusory) numerosity and discuss their findings based on earlier research suggesting critical stages of numerosity perception around 150 ms in V3^62^. Interestingly, also in other domains of visual perception, research on reward effects indicates early modulations of bottom-up attention^63^ that are also associated with signalling in the early visual cortex^64,65^. Further research is necessary to better understand perceptual mechanisms underlying the modulation of the pupillary response during incentivized numerosity discrimination.

In sum, the present study shows that incentive motivation can enhance ANS precision in a non-symbolic dot comparison task in children and adolescents. Applying the EZ-diffusion model allowed us to separate reward effects and ageing effects on distinct components of the perceptual decision-making process, like modulations in perceptual efficiency (i.e. drift rate) and response caution (i.e. boundary separation). We found steeper drift rates in both age groups in rewarded trials. As an indicator of dopaminergic reward processing, we assessed changes in pupil diameter. In both age groups, we found greater pupil dilations in rewarded trials in all three trial phases. Resting upon earlier evidence on the association between pupil dilation and activity in the salience network of the brain during reward anticipation^33^, our results suggest that the reward effect on ANS precision may be based on a dopaminergic mechanism in which reward increases the perceptual salience of reward-associated stimuli, thereby directly affecting perception. Future research combining behavioural data on numerosity perception with structural brain measures could help to substantiate this interpretation. From a methodological point of view, follow-up studies should implement an inter-stimulus- and inter-trial interval to avoid overlapping of processes between trial phases as well as confounding effects of earlier processes on the pupillary response^66^. Further, future studies could use a reward scheme that takes both into account, participants’ speed and accuracy. From an educational perspective, it would be worthwhile to examine if incentive modulations of ANS precision are reflected in improved symbolic math performance. Symbolic mathematic proficiency has been related to the ANS^6,67^, although evidence on this link has been challenged^68^ and a recent meta-analysis suggests only a weak non-significant effect of ANS training on symbolic math performance^69^. However, the mechanisms underlying these training studies, which rely on accuracy-feedback only, have not been considered in this context. Finally, we can summarize that the assumptions of the Incentive Salience Hypothesis probably apply to the perception of numerosities also in younger subjects.

## Methods

### Participants

A total of 57 7-year-old children (*n* = 27; all White) and 14-year-old adolescents (*n* = 30; all White) residing in Dresden and its environs took part in this study. This sample was chosen as (1) it allows drawing inferences on children at the beginning of their formal education and youth after puberty should have started, and (2) cross-sectional designs with narrow age-cohort samples as compared to age-heterogeneous designs are more sensitive to condition effects^70^. Due to severe signal loss during pupil tracking resulting in noisy data for four children (see Data Analyses), the final sample consisted of 23 children (12 girls) and 30 adolescents (15 girls) in the analyses. An a priori power calculation indicated sufficient power with a reasonable low probability of a type II error and a high likelihood for detecting potential effects of reward in a sample of this size (required total sample size of *N* = 50; see Supplementary Methods for more details).

All participants had normal or corrected-to-normal vision, no history of neurological or psychiatric diseases, and were not taking any medication. Further psychometric information is provided in the supplementary material. The study was approved by the ethics committee of the TU Dresden (EK 55,022,017) in accordance with the Declaration of Helsinki and performed following the relevant guidelines and regulations. The parents of the children and adolescents gave their written informed consent prior to study participation. After the testing, adolescents received 15 and children received 25 Euro for their participation—testings for children took longer and, therefore, were realized in two separated sessions (see more details below), where the payment for the two age groups translates to about equal hourly reimbursement (10 EUR per hour in the lab, and 5 EUR for travelling)—plus the bonus they earned during the incentivized non-symbolic dot comparison task (i.e. prizes with an average value of 10.33 Euro for the entire sample; a mean value of 10.05 Euro in the children group and a mean value of 10.54 Euro in the adolescent group). The distribution of different win options (see the following section for more details) did not differ across age groups, χ^2^(3, *n* = 53) = 0.52, *p* = 0.92.

### Experimental paradigm

Participants performed an incentivized non-symbolic dot comparison task (see Fig. 1a and elsewhere^11^). In each trial of this task, two dot clouds are presented on a computer screen and participants have to identify the cloud with more dots. First, a pink or blue fixation cross (luminance: 1.17 cd/m^2^), the incentive cue, appears in the centre of the screen for 1500 ms. The colour of the cue signals the incentive condition (colour assignment counterbalanced across participants). In trials of the reward condition but not of the control condition, each correct or error response resulted in gaining or losing three points, respectively. The incentive cue is followed by the task stimulus, two dot clouds that are flanking a grey fixation cross (luminance: *M* = 0.97 cd/m^2^) and disappear after participants make their decision or after 1200 ms at the latest. Participants were instructed to decide as quickly and accurately as possible by pressing the left or the right control key on a keyboard. Responses were accepted until 3000 ms after stimulus onset. Finally, participants received accuracy feedback based on a happy or sad emoticon (luminance: 1.17 cd/m^2^) together with the information about points earned or lost indicated by a number (± 3 in the reward condition, 0 in the control condition), both shown for a period of 1500 ms. Trial types (reward vs. control) were randomized within blocks.

During a short break of ten seconds between blocks, participants were shown information on the thus far achieved bonus level and associated win. In total, there were 15 bonus levels and five possible win options. Participants could reach a higher bonus level by earning points during reward trials of the task. Scores from an earlier pilot study with children and adolescents of the same age were used to set the bonus levels and win options. More precisely, to achieve a comparable reward scheme for children and adolescents the bonus levels were defined based on the percentile ranks (*PR*) within each age group resulting in the following threshold values for the five win options: a pen at *PR* = 0.05 (1) or a voucher for a local shopping mall worth 10 Euro at *PR* = 35 (2), 15 Euro at *PR* = 85 (3), 20 Euro at *PR* = 95 (4), or 25 Euro at *PR* = 99.9 (5). Bonus information provided in the first five seconds of the break between blocks referred to the, thus far, accumulated points, the current bonus level reached, and the distance to the next level (see Fig. 1b) and were followed by a countdown to prepare participants for the start of the first trial of the next block. The task consisted of 32 blocks with 24 trials each and took about 50 to 60 min to complete.

Participants received instructions and four practice trials were conducted before they performed the test blocks. Adolescents received written instructions presented in white letters on a black background. Children received oral instructions along with coloured illustrations on a black background. The dot arrays and the feedback at the end of each trial were grey and displayed on a black background as well. The maximum field area of the dot arrays encompassed 7.5° visual angles in diameter. The number of dots in each array varied between 12 and 32, with a ratio between the two arrays of 4:3 (3:4), 5:4 (4:5), 8:7 (7:8) or 10:9 (9:10). This procedure was based on earlier studies with children^5,67^ and adults^23^. Further details on data acquisition as well as on stimulus generation and procedures of experiment controls are described in the Supplementary Methods and elsewhere^11^, where the same experimental paradigm was used to study the impact of incentive motivation on numerosity discrimination in young adults. Likewise, in the present study, we measured RTs, error rates and PD during the task as dependent variables.

### Data analyses

We used the statistical software package IBM SPSS Statistics for Windows, Version 27.0 (IBM Corp., Armonk, NY, USA) and R, Version 3.6.0^71^ in R Studio 1.2.1335 (RStudio, Inc.) for the analysis of the behavioural data (RTs, accuracy). First, outlier trials and items were removed (for more details, see Supplementary Methods and information below). After the exclusion of all outliers, mean RTs and accuracy (i.e. the proportion of correct responses) were computed for each condition and participant. Further, an EZ-diffusion model was applied to the data in order to dissect the decision-making process^42^. For this, trials with RTs shorter than 250 ms or longer than 1500 ms had to be excluded^72^, which we also describe in more detail in the Supplementary Methods. Next, based on the accuracy data as well as the mean and variance of RTs of correct responses three parameters per condition per participant were estimated: drift rate (v), boundary separation (a) and non-decision time (t_ER_). Importantly, a potential underestimation of the drift rate in the group of adolescents needs to be taken into account when interpreting the results^42^ as tests regarding the suitability of the data for the application of the EZ-diffusion model suggest (see Supplementary Methods for more details).

Matlab 9.6.0, R2019B (The MathWorks, Inc., MA, USA), SPSS 27 and R, Version 3.6.0 in R Studio 1.2.1335 were used for the processing of the pupillary data. First, standard procedures were used to clean the pupillary data, the average pupil diameter of the left and right eye^73^. Trials with excessive blinking were discarded and small blinks replaced by cubical interpolation. The data of the children showed more artefacts compared to the adolescents’ data. Due to noisy data, four of the originally 27 children had to be excluded from further analyses. After discarding outliers and artefacts 55.04% of all trials (reward: 53.69%; control: 56.40%) remained, but only 46.29% for the children compared to 61.75% for the adolescents. We assume this great loss of data to result mainly from motion artefacts as we did not stabilize participants’ heads. As pupil measures can be affected by gaze shifts distorting the shape of the pupil (i.e. parallax; see^74^), we analysed participants’ gaze position for the remaining data. Parallax effects are minimal when gaze shifts are small (less than ~ 10°). Therefore, the percentage of gaze time spent within a central area on the screen smaller than 10° visual angle in diameter was calculated per trial. Only gaze position during fixations and saccades were considered, while for 4.48% of the data (children: 3.97%; adolescents: 4.94%) no gaze event could be determined due to blinks or other artefacts. Afterwards, means per participant and condition (reward vs. control) during the presentation of the cue, the stimulus and the feedback were determined and subjected to a mixed ANOVA with Incentive (reward vs. control) and Phase (cue, stimulus, feedback) as within-subject factors and Age Group (children vs. adolescents) as between-subject factor. Results show that participants spent more than 97% of the time looking at the central area on the screen. There was no difference between age groups (*F*(1,51) = 1.06, *p* = 0.31, η_p_^2^ = 0.02) nor between conditions (*F*(1,51) = 1.98, *p* = 0.17, η_p_^2^ = 0.04) except during the processing of the feedback, where gaze time within the central area was longer in trials of the control condition (97.55%) compared to rewarded trials (97.17%), as a significant interaction Incentive × Phase (*F*(1.33,68.02) = 7.80, *p* < 0.01, η_p_^2^ = 0.13) and pairwise comparisons suggest (*p* = 0.01). Due to the low proportion of larger gaze shifts (i.e. gaze time outside the central area), this difference might be negligible, and altogether the eye movement analysis corroborates the reliability and comparability of the pupillometry data.

Following this, trials were separated into three phases: reward anticipation (cue phase), numerosity discrimination (stimulus phase) and outcome (feedback phase). The different phases were analysed individually. For each condition and participant, we computed a stimulus-locked pupillary response for the cue and the feedback phase (length: 1500 ms after cue or feedback presentation) and a response-locked pupillary response for the stimulus phase (length: 1500 ms until 200 ms after button press). The data was smoothed by an unweighted 5-point moving average filter. Further, for each trial, a baseline correction was done by computing the mean pupil diameter of a 200 ms period preceding the phase-onset, which then was subtracted from the pupillary response to depict the phase-related PD. Afterwards, the response curves for all valid trials were averaged per condition per participant, each average response curve calculated from a minimum of 10 trials to have a reliable measurement with sufficiently reduced noise (median of 219 trials; children: 125 trials; adolescents: 238 trials). The peak PD (i.e. the maximal PD) was entered into the statistical analysis of each phase. In the cue phase, the peak was defined as the dilation relative to the minimum of the pupil diameter as baseline correction resulted in negative values and a maximum often at the beginning of the trial. At this time and other than in the stimulus and feedback phase, the pupil was still recovering due to the missing inter-trial interval (cf. Fig. 4a).

Linear mixed effects models were conducted using the lme function from the nlme package in R^75^ with maximum-likelihood estimation. Subjects were entered as random intercepts and a within-subject effect of the factor Incentive (i.e. reward vs. control condition) and a between-subject effect of the factor Age Group (i.e. children vs. adolescents) was analysed for performance measures (RTs, accuracy), the parameter estimates of the EZ-diffusion model (v, a, t_ER_) and the peak PD separately for the three different trial phases (cue, stimulus, feedback). Similar to the earlier study on young adults^11^, we also conducted linear mixed effects models considering the factor Ratio (i.e. four ratio conditions) as within-subject effect for all measures. For these analyses, three children and two adolescents had to be excluded due to the reduced trial number per condition and corresponding noisy pupillary data. Results are reported in the supplementary material and show that the effect of Ratio is similar to earlier findings we observed in young adults with worse performance (longer RTs, lower accuracy), a less efficient and careful decision process (lower drift rate and boundary separation, longer non-decision time) and more effortful processing (larger PD in the stimulus and feedback phase) for lower ratios (i.e. higher difficulty) in both age groups^11^. Results regarding the effects of Incentive and Age Group are similar to the results of the simpler models without the factor Ratio. Given the focus of the current study and for reasons of clarity the Results section reports the findings of these simpler models only. Following the approach and recommendations by Fern and Monroe^76^ and Maxwell, et al.^77^, we report partial eta squared (η_p_^2^) for effect sizes. Pairwise t-tests were performed for posthoc multiple-comparison with Holm-correction to correct for family-wise error^78^. The Saphiro-Wilk-test as well as Q-Q-plots revealed that the residuals of some models were not normally distributed. In this case, permutation tests using the lmer function from the lme4 package^79^ and permanova from the predictmeans package in R^80^ were carried out. As these tests showed comparable results, we only report the results of the linear mixed effects models. All analyses were confirmatory testing of the hypothesis deducted in the introduction. A rejection criterion of *p* ≤ 0.05 (two-tailed; if not stated differently) was chosen for all statistical tests.

## Supplementary Information

Supplementary Information.

## Data Availability

The datasets generated and analyzed during the current study are not publicly available due to privacy and ethical restrictions but are available from the corresponding author on reasonable request.
