# Supplementary material for: Incentive motivation improves numerosity discrimination in children and adolescents
Source: Sci Rep. 2022 Jun 16;12:10038. doi: 10.1038/s41598-022-14198-7 (PMC9203779; doi:10.1038/s41598-022-14198-7)
Supplement: Supplementary file 1 — Supplementary Information. [file 41598_2022_14198_MOESM1_ESM.docx]

**Incentive motivation improves numerosity discrimination in children and adolescents**

Luca Spliethoff, Shu-Chen Li, and Annika Dix

**Supplementary Results**

Descriptive statistics are displayed in Table S1, including means and standard deviations of the behavioural performance (RTs, accuracy), the model parameters of the decision-making process (drift rate, boundary separation, and non-decision time) as well as the peak PD in the three trial phases (cue phase, stimulus phase, feedback phase) separated for the two incentive conditions (reward vs. control) and the two age groups (children vs. adolescents).

***Table S1.*** Descriptive statistics: mean (*M*) and standard deviation (*SD*) for the behavioural performance measures, the model parameters of the decision-making process, and the pupillometry measures.

| **Age Group** | **Reward** | | **Control** | | |  |
| --- | --- | --- | --- | --- | --- | --- |
|  | **Children** | **Adolescents** | | **Children** | **Adolescents** | |
| RTs | | | | | |  |
| *M* (ms) | 915.79 | 699.02 | | 936.40 | 686.75 | |
| *SD* (ms) | 136.29 | 138.73 | | 157.26 | 114.37 | |
| Accuracy | | | | | |  |
| *M* (%) | 74.14 | 83.00 | | 71.37 | 80.98 | |
| *SD* (%) | 6.39 | 4.23 | | 7.49 | 4.09 | |
| Drift rate (v) | | | | | |  |
| *M* | 0.12 | 0.17 | | 0.10 | 0.16 | |
| *SD* | 0.03 | 0.03 | | 0.04 | 0.03 | |
| Boundary separation (a) | | | | | |  |
| *M* | 0.10 | 0.09 | | 0.10 | 0.09 | |
| *SD* | 0.01 | 0.01 | | 0.01 | 0.02 | |
| Non-decision time (t_ER_) | | | | | |  |
| *M* | 0.68 | 0.50 | | 0.66 | 0.49 | |
| *SD* | 0.10 | 0.08 | | 0.13 | 0.06 | |
| Peak pupil dilation – cue phase | | | | | |  |
| *M* (mm) | 0.03 | 0.07 | | 0.02 | 0.04 | |
| *SD* (mm) | 0.04 | 0.05 | | 0.03 | 0.04 | |
| Peak pupil dilation – stimulus phase | | | | | |  |
| *M* (mm) | 0.15 | 0.21 | | 0.13 | 0.16 | |
| *SD* (mm) | 0.09 | 0.09 | | 0.10 | 0.08 | |
| Peak pupil dilation – feedback phase | | | | | |  |
| *M* (mm) | 0.25 | 0.24 | | 0.22 | 0.19 | |
| *SD* (mm) | 0.09 | 0.08 | | 0.078 | 0.08 | |

The linear mixed effects models integrating the factor Ratio as fixed effects revealed similar effects of Incentive and Age Group as shown in Table S2 for behavioural performance (RTs, accuracy), in Table S3 for the model parameters of the decision-making process (drift rate, boundary separation, and non-decision time), and in Table S4 for the peak PD in the three trial phases (cue phase, stimulus phase, feedback phase). Further, similar to earlier findings on the effect of Ratio we observed in young adults^1^, in the present study lower ratios (i.e. higher difficulty) were associated with worse performance (longer RTs, lower accuracy), a less efficient and careful decision process (lower drift rate and boundary separation, longer non-decision time) and more effortful processing (larger PD in the stimulus and feedback phase) in both age groups. The results for the different parameters separated for the two incentive conditions (reward vs. control), the four ratio conditions and the two age groups (children vs. adolescents) are illustrated in Figure S1, Figure S2 and Figure S3 correspondingly.

***Table S2.*** Statistical overview on the main and interaction effects of the linear mixed effects models with subjects as random intercepts and the factors *Age Group* (children vs. adolescents), *Incentive* (reward vs. control) and *Ratio* (four ratio conditions) on response times and accuracy.

| **Factor** | **Response Times** | | **Accuracy** | |
| --- | --- | --- | --- | --- |
|  | *F* statistic | *p*-value | *F* statistic | *p*-value |
| Intercept | 1599.32 | < .0001 | 12008.36 | < .0001 |
| Age Group | 31.31 | < .0001 | 44.71 | < .0001 |
| Incentive | 0.20 | 0.66 | 31.36 | < .0001 |
| Ratio | 43.20 | < .0001 | 324.79 | < .0001 |
| Age Group × Incentive | 17.71 | < .0001 | 3.00 | 0.08 |
| Age Group × Ratio | 0.96 | 0.41 | 2.49 | 0.06 |
| Incentive × Ratio | 1.14 | 0.33 | 1.31 | 0.27 |
| Age Group × Incentive × Ratio | 1.30 | 0.28 | 0.66 | 0.58 |

***Table S3.*** Statistical overview on the main and interaction effects of the linear mixed effects models with subjects as random intercepts and the factors *Age Group* (children vs. adolescents), *Incentive* (reward vs. control) and *Ratio* (four ratio conditions) for the model parameters of the decision-making process.

| **Factor** | **Drift Rate (v)** | | **Boundary Separation (a)** | | **Non-Decision Time (t_ER_)** | |
| --- | --- | --- | --- | --- | --- | --- |
|  | *F* statistic | *p*-value | *F* statistic | *p*-value | *F* statistic | *p*-value |
| Intercept | 1153.12 | < .0001 | 2085.04 | < .0001 | 1883.19 | < .0001 |
| Age Group | 64.85 | < .0001 | 1.08 | .30 | 38.06 | < .0001 |
| Incentive | 39.44 | < .0001 | 4.47 | .04 | 17.89 | < .0001 |
| Ratio | 302.15 | < .0001 | 22.74 | < .0001 | 20.06 | < .0001 |
| Age Group × Incentive | 1.19 | .28 | 8.79 | < .01 | 1.03 | .31 |
| Age Group × Ratio | 14.15 | < .0001 | 1.89 | .13 | 0.52 | .67 |
| Incentive × Ratio | 0.39 | .76 | 0.26 | .86 | 0.96 | .41 |
| Age Group × Incentive × Ratio | 0.75 | .53 | 1.37 | .25 | 0.51 | .67 |

***Table S4.*** Statistical overview on the main and interaction effects of the linear mixed effects models with subjects as random intercepts and the factors *Age Group* (children vs. adolescents), *Incentive* (reward vs. control) and *Ratio* (four ratio conditions) for the peak PD in the three trial phases (cue phase, stimulus phase, feedback phase).

| **Factor** | **PD - Cue** | | **PD - Stimulus** | | **PD - Feedback** | |
| --- | --- | --- | --- | --- | --- | --- |
|  | *F* statistic | *p*-value | *F* statistic | *p*-value | *F* statistic | *p*-value |
| Intercept | 80.97 | < .0001 | 207.21 | < .0001 | 439.68 | < .0001 |
| Age Group | 3.03 | .09 | 2.02 | .16 | 0.90 | .35 |
| Incentive | 65.82 | < .0001 | 90.04 | < .0001 | 69.96 | < .0001 |
| Ratio | 1.36 | .26 | 4.61 | < .01 | 3.00 | .03 |
| Age Group × Incentive | 3.81 | .05 | 9.92 | < .01 | 2.29 | .13 |
| Age Group × Ratio | 0.14 | .94 | 0.60 | .62 | 1.02 | .38 |
| Incentive × Ratio | 0.37 | .78 | 0.69 | .56 | 0.23 | .87 |
| Age Group × Incentive × Ratio | 1.20 | .31 | 0.25 | .86 | 0.19 | .90 |


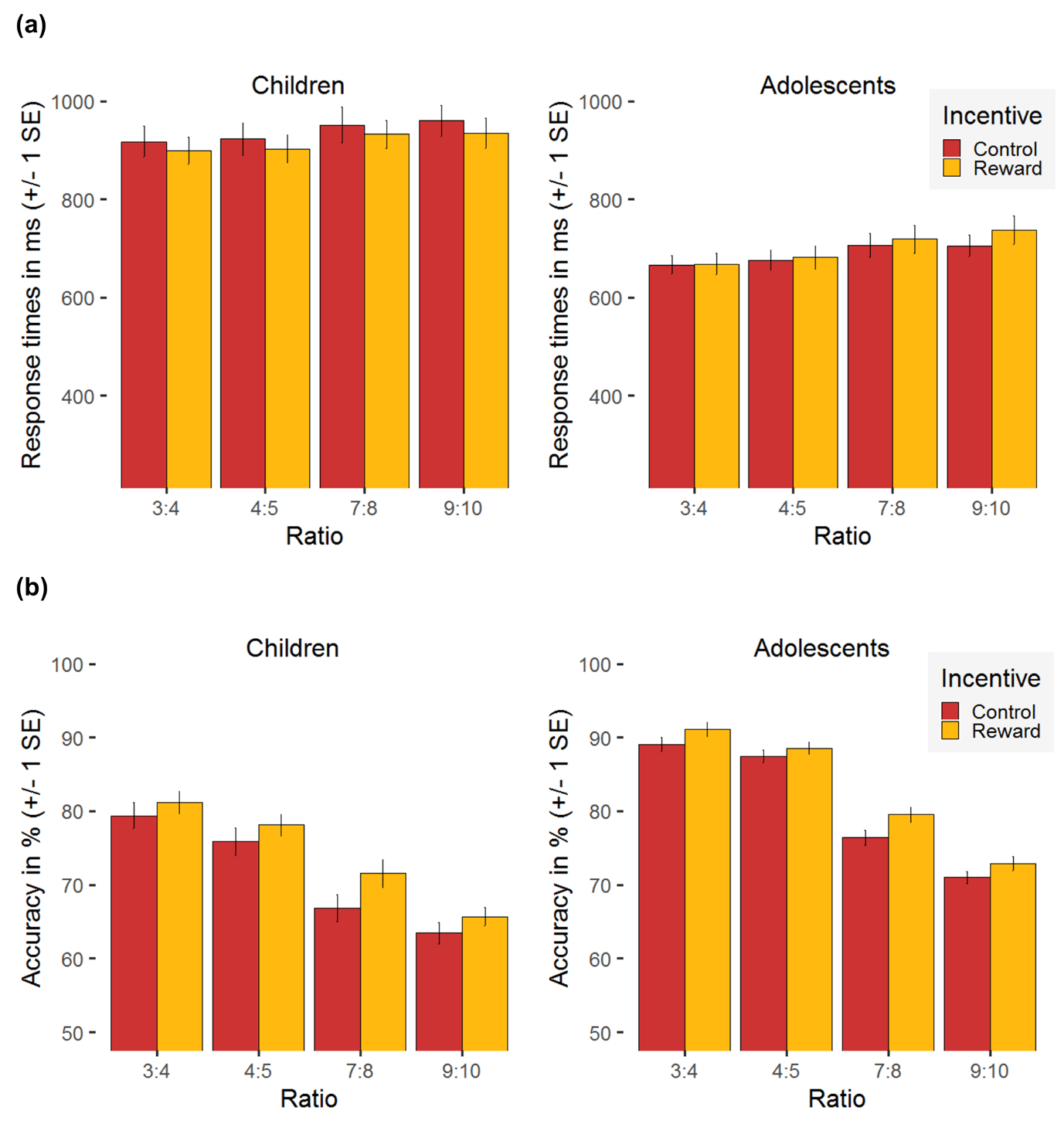


***Figure S1.*** Behavioural performance: Mean and standard error (SE) for **(A)** response times in milliseconds (RT) and **(B)** accuracy in percent, both separated for the two incentive conditions (reward vs. control), the four ratio conditions, and the two age groups (children vs. adolescents).


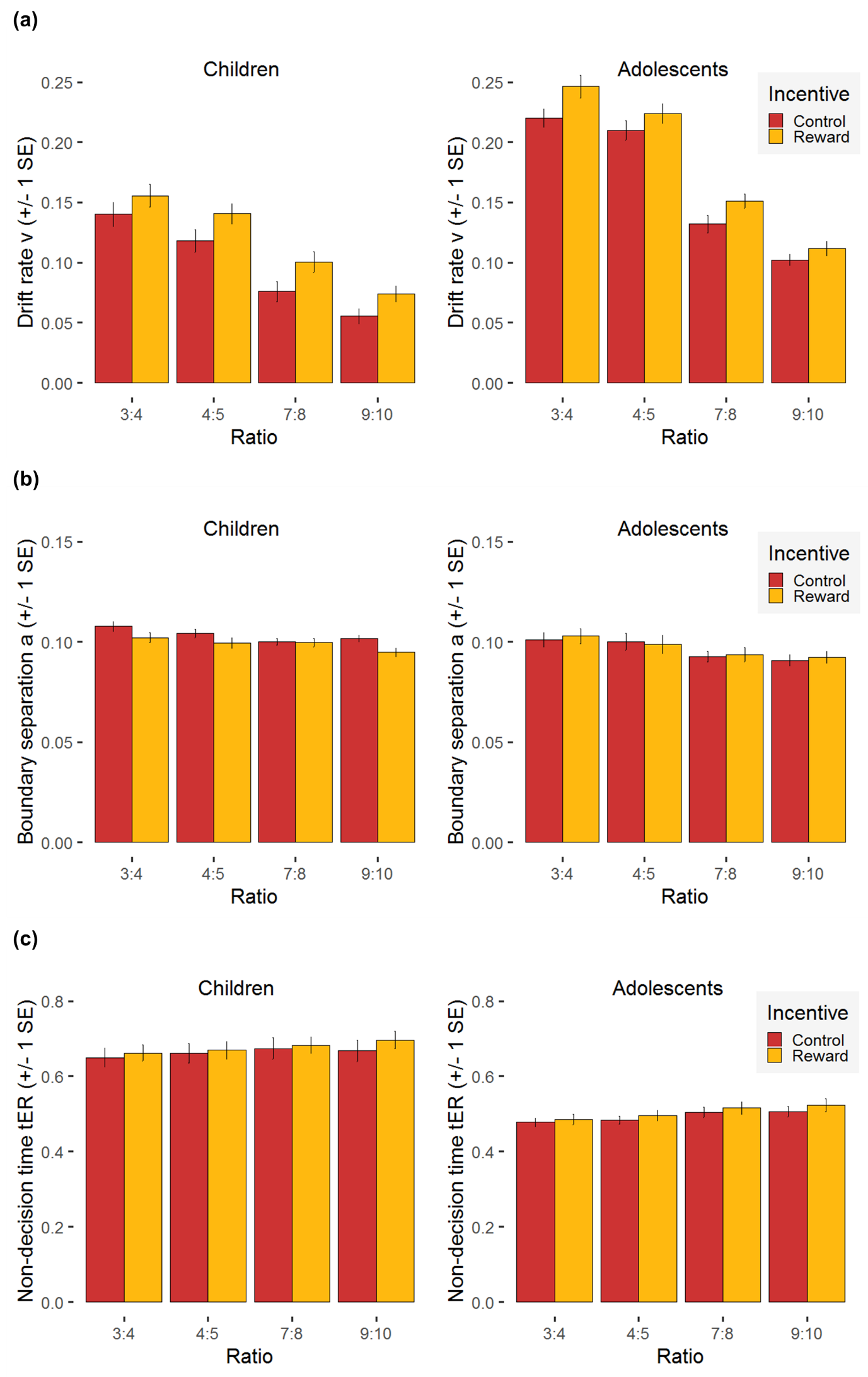


***Figure S2.*** Model parameters of the decision-making process: Mean and standard error (SE) for **(A)** drift rate (v), **(B)** boundary separation (a), and **(C)** non-decision time (t_ER_), separated for the two incentive conditions (reward vs. control), the four ratio conditions, and the two age groups (children vs. adolescents).


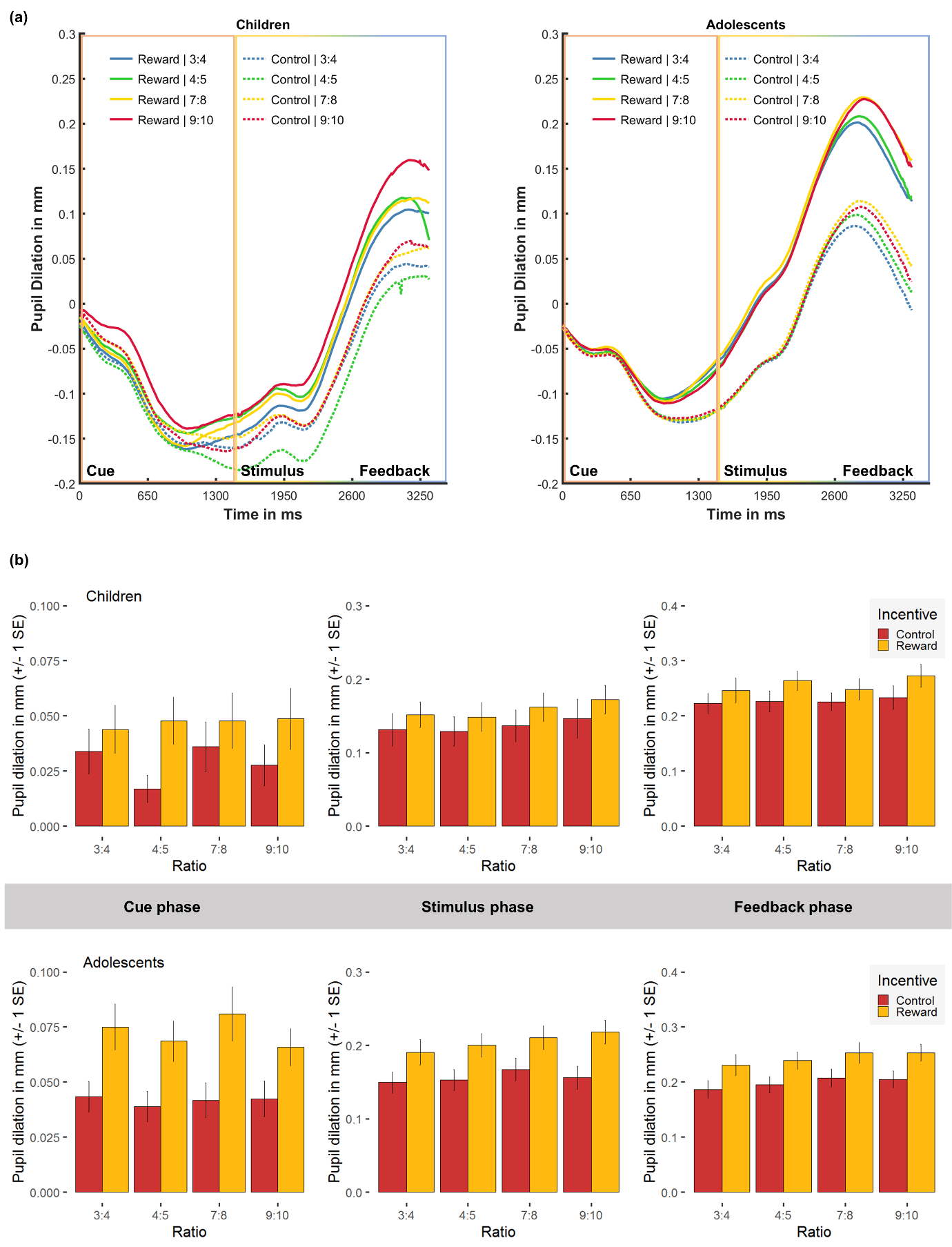


***Figure S3.*** Pupillometry measures: **(A)** Mean pupil response (pupil dilation in millimetre) for children (left) and adolescents (right) across the time course of the trial (in milliseconds) with the associated phase, stimulus-locked to the onset of the cue (zero point in time) and separated for the different conditions (incentive condition: reward vs. control; four ratio conditions); **(B)** mean and standard error (SE) for the pupil dilation (in millimetres) measured at the peak of the pupil response in children (top) and adolescents (bottom) during reward anticipation (cue phase; left), numerosity discrimination (stimulus phase; middle), and processing of the outcome (feedback phase; right), separated for the two incentive conditions and the four ratio conditions.

**Supplementary Methods**

**Participants**

The a priori power calculation indicated sufficient power with a reasonable low probability of a type II error and a high likelihood for detecting potential effects of reward for the size of the sample in the present study (required total sample size of *N* = 50). It was based on prior knowledge of the following: (1) the impact of reward on the performance in an earlier version of the incentivized non-symbolic dot comparison task we validated in a pilot study with children and adolescents; and (2) changes in peak pupil dilation in response to reward we could show in a sample of young adults during numerosity discrimination (elsewhere). The power calculation using G*Power 3.1 ^2^ was based on the smallest effect referring to the interaction between age group and reward, which we observed in the behavioural pilot study, with the following input parameters: an effect size of f = 0.20 with two measurements (reward vs. control), a correlation of *r* = 0.50 between these two, a significance level of α = 0.05 and the statistical power 1-β = 0.80.

Both groups underwent a psychometric assessment. Table S5 shows all psychometric information including measures of participants’ math anxiety (Modiefied Abbreviated Math Anxiety Scale, mAMAS ^3^), motivational systems for behavioural inhibition and activation Behavioural Inhibition/ Behavioural Activation System Scales, BIS/BAS ^4^), need for cognition (Need for Cognition Scale for Children and Adolescents, NFC ^5^), performance on a symbolic numerical discrimination test ^6^ and a mathematic achievement test (Calculation Subtest of the Woodcock-Johnson-Revised Tests of Achievement, WJ-R ^7^). perception speed (Identical-Pictures Test ^8^), vocabulary using age-appropriate tests for children (subtest of Wechsler Intelligence Scale for Children, WISC-IV ^9^) and adolescents (Mehrfach-Wahl-Wortschatz-Test B, MWT-B ^10^) and spatial working memory ^11^.

***Table S5.*** Descriptive statistics: mean (*M*) and standard deviation (*SD*) or frequency (*f*) for the psychometric measures.

|  | | **Children** | | | | **Adolescents** | | |
| --- | --- | --- | --- | --- | --- | --- | --- | --- |
|  |  | ***M*** | | ***SD*** | | ***M*** | | ***SD*** |
|  | | ***f*** | | | | ***f*** | | |
| mAMAS | | 14.22 | | 4.69 | | 19.97 | | 5.17 |
| BIS | | 2.51 | | 0.59 | | 2.74^e^ | | 0.44 |
| BAS^a^ | |  | |  | |  | |  |
| Drive | | 3.15 | | 0.57 | | 2.90^e^ | | 0.44 |
| Fun seeking | | 3.32 | | 0.48 | | 2.85^e^ | | 0.62 |
| Reward | | 3.39 | | 0.65 | | 3.12^e^ | | 0.49 |
| NFC^b^ | |  | |  | |  | |  |
| Think | | 32.57 | | 5.80 | | 31.17 | | 4.82 |
| Seek | | 15.96 | | 3.72 | | 14.43 | | 3.17 |
| Conquer | | 10.09 | | 1.73 | | 9.87 | | 1.46 |
| Symbolic NDT^c^ | |  | |  | |  | |  |
| Error prevalence (%) | | 47.80 | | | | 13.3 | | |
| RT (s) | | 87.43 | | 15.87 | | 0.66 | | 0.49 |
| WJ-R | | 10.17 | | 2.31 | | 56.84^e^ | | 181.47 |
| Vocabulary^d^ | | 24.05^f^ | | 6.20 | | 9.90 | | 5.06 |
| Identical-Pictures Test | |  | |  | |  | |  |
| RT (ms) | | 4912,00 | | 1484.78 | | 2320.77 | | 351.74 |
| Correct responses | | 14.22 | | 4.10 | | 29.69 | | 4.42 |
| Spatial WM | |  | |  | |  | |  |
| Recognition accuracy (%) | | 73.01 | | 14.52 | | 92.85 | | 6.98 |
| Recall accuracy (%) | 18.62 | | 23.93 | | 88.31 | | 18.15 | |

^a^ Subscales of BAS: Drive (i.e. motivation to follow one’s goals), Fun Seeking (i.e. motivation to find novel rewards spontaneously), and Reward Responsiveness (i.e. sensitivity to pleasant reinforcers)

^b^ Subscales of NFC: general factor Think as well as specific factors Seek (i.e. approaching intellectually challenging situations) and Conquer (i.e. expending effort to master these challenges)

^c^ Symbolic numerical discrimination test

^d^ As different tests were used for children and adolescents, mean scores are not comparable between groups

^e^ Missing data for one participant resulting in a group sample size of *n* = 29 for adolescents

^f^ Missing data for one participant resulting in a group sample size of *n* = 22 for children

**Experimental Paradigm**

The dot size of the stimuli varied between 2.66 mm and 5.61 mm. The dots were isoluminant for all trials, the pixel intensity was set to 73 on an 8-bit gray scale. The position (left vs. right) of the larger dot array was randomly assigned and each stimulus was presented twice in the course of the experiment, once in the reward condition and once in the control condition. Further, an orthogonal design was used to match the number of dots and the ratio between the arrays (see Table S5). Accordingly, the number of dots was not informative about the ratio between the arrays or vice versa and the visual characteristics such as pixel intensity or contrast of the stimuli were comparable between ratios. Additionally, to encourage participants to make their decisions on the dimension of number we controlled the visuospatial dimensions of size and spacing of the dot arrays following the approach by DeWind, et al. ^12^. Thereby, dot size vs. total surface area, on the one hand, and sparsity vs. total field area, on the other hand, were matched between the dot arrays. This also reduced the impact of different confounds on pupil size like luminance or density. For both dimensions (i.e. size and spacing), we controlled for the one aspect (e.g., dot size) in one half of the trials and for the other aspect (e.g., total surface area) in the other half of the trials. Controls of the two dimensions were randomly and independently chosen across trials.

***Table S6.*** Numerosities of the stimuli using six different pairings varying in quantity (increasing numerosity from 1 to 6) with two presentation orders each (larger numerosity on the left vs. right) separated for the four ratio conditions.

| **Pairing** | **Ratio** | | | | | | | |
| --- | --- | --- | --- | --- | --- | --- | --- | --- |
|  | **4:3 / 3:4** | | **5:4 / 4:5** | | **8:7 / 7:8** | | **10:9 / 9:10** | |
| 1 | 12 | 16 | 12 | 15 | 12 | 14 | 12 | 13 |
|  | 16 | 12 | 15 | 12 | 14 | 12 | 13 | 12 |
| 2 | 13 | 17 | 13 | 16 | 13 | 15 | 13 | 14 |
|  | 17 | 13 | 16 | 13 | 15 | 13 | 14 | 13 |
| 3 | 18 | 24 | 19 | 24 | 18 | 21 | 19 | 21 |
|  | 24 | 18 | 24 | 19 | 21 | 18 | 21 | 19 |
| 4 | 19 | 25 | 20 | 25 | 19 | 22 | 20 | 22 |
|  | 25 | 19 | 25 | 20 | 22 | 19 | 22 | 20 |
| 5 | 21 | 28 | 21 | 26 | 21 | 24 | 21 | 23 |
|  | 28 | 21 | 26 | 21 | 24 | 21 | 23 | 21 |
| 6 | 22 | 29 | 23 | 29 | 27 | 31 | 27 | 30 |
|  | 29 | 22 | 29 | 23 | 31 | 27 | 30 | 27 |

**Data Acquisition**

Individual testings were conducted for each participant and took place at the laboratory of the chair in a quiet moderately illuminated room (background luminance ca. 350 lx). First, participants, if necessary together with their parents (i.e. for children), completed a paper-and-pencil questionnaire on pupil-influencing factors (e.g., drug consumption, medication, psychiatric and neurological dysfunction; cf. elsewhere ^13^). Afterwards, the experimental task (i.e. the incentivized non-symbolic dot comparison task) was performed on a computer using the software Presentation 18.1 (Neurobehavioral Systems Inc, Albany, CA) running on a Microsoft® Windows® 7 operating system, which recorded the behavioural data. The computer screen (size of the display: 23, display resolution: 1680 × 1050) was placed at a distance of 65 cm. A second computer recorded the measurement of the pupil diameter using the software Tobii Studio 3.4.5 (Tobii Technology AB, Stockholm, Sweden). The Tobii Workspace Extension for Presentation was employed to control the pupil recording software with the first computer. An infrared binocular eye tracking system, a Tobii Pro TX300 (Tobii Technology AB, Stockholm, Sweden), tracked the pupil diameter (left and right eye in millimetres) with dark pupil tracking and a sampling frequency of 300 Hz. Participants could move the head naturally in front of the computer screen, to which the eye tracking unit was attached. In preparation for the testing, a 9-point calibration and a five minutes gaze recording, during which a grey fixation cross on a black background (1.17 cd/m²) had to be fixated, was conducted as we also recorded gaze data (accuracy: 0.4° of visual angle) and participants’ spontaneous eye blinkrate ^14^. The psychometric assessment (paper-and-pencil tests and computer-based measures) was conducted before or after the incentivized non-symbolic dot comparison task was completed (counterbalanced across participants), together with a second experimental task, a non-symbolic arithmetic task adopted from Hyde, et al. ^15^ as well as Park and Brannon ^16^. Finally, participants were compensated for their participation and received the prize they won in the incentivized dot comparison task.

**Data Analyses**

Outlier trials were defined as correct trials with RTs deviating 3.5 standard deviations (SDs) from the individuals’ mean, and items, for which RTs deviated 3.5 SDs from the group mean of the regarded condition (reward vs. control). Thereby, in the group of children, 2.14% of the trials and in the group of adolescents, 0.80% of the trials were eliminated. The prevalence of outliers was independent of the condition in both age groups. Though more trials were excluded for children, the final item pool considered for the statistical analysis did not differ between groups. In total five items were identified as outliers and excluded consistently for both age groups. To apply an EZ-diffusion model, which requires the exclusion of trials with RTs shorter than 250 ms or longer than 1,500 ms, another 5.45% of the trials for children and 1.16% of the trials for adolescents were eliminated. Overall, this affected the number of trials considered for the analyses similarly in both conditions. However, for children more correct and incorrect long trials (RT > 1,500 ms) had to be excluded in the control compared to the reward condition, χ²(1, *N* = 21706) = 11.34, *p* = 0.001. For adolescents, conversely, more correct and incorrect long trials had to be excluded in the reward compared to the control condition, χ²(1, *N* = 23618) = 11.39, *p* = 0.001. We do not assume that this could have changed the findings considerably. If at all, this might have weakened the effects we found regarding the decision-making process (cf. the impact of incentive and age on the model parameters of the decision-making process, particularly the boundary separation, described in the Results section).

The application of the EZ-diffusion model requires ex-Gaussian distribution of the RT data ^17^. The key characteristics of the RT distributions (mean, variance, kurtosis and skewness) indicate suitability for the chosen model approach with only a few exceptions (RT distribution in the control condition for two children and one adolescent). Further, the starting point of the model has to be equidistant from the two response boundaries, that is, both response options should be equally attractive. Therefore, the relative speed of correct and error responses for the different target positions of the larger dot array was compared. ANOVAs with Target (left vs. right) and Response (correct vs. error) as within-subject factors and RTs as the dependent variable were computed for each condition and each participant. Though a violation of this requirement was thought to be unlikely as the paradigm does not favour one response (left vs. right) over the other (e.g., due to different presentation rates), results for two children (reward and/or control condition) point to a potential prioritization of one answer button (not corrected for family-wise error). After excluding the four children and one adolescent, for which data does not meet the requirements stated above, results of the linear mixed effects models for the parameter estimates did not change considerably and, therefore, findings for the complete sample were reported. To further check for potential misspecifications of the model, the RT distributions of correct and error responses were compared. In 53 out of 106 cases (reward: eight children, 23 adolescents; control: eight children, 14 adolescents), Mann-Whitney-U test revealed that RTs were significantly faster for correct compared to error responses (without correction for family-wise error), which is an indicator of across-trial variability in drift rate. In addition, in another five cases in the control condition (two children and three adolescents) RTs were significantly slower for correct compared to error responses (without correction for family-wise error), which is an indicator of across-trial variability in the starting point. Both aspects cannot be considered by the EZ-diffusion model and it has been shown that the EZ-diffusion model underestimates all parameters, particularly the drift rate, in such cases ^17^. Therefore, the resulting underestimation of the parameters, predominantly in the group of adolescents, should be taken into account for their interpretation.

In the Supplementary Material, we also report results for the analyses integrating the factor Ratio. For the application of the EZ-diffusion model, the characteristics of the RT distribution indicate ex-Gaussian distributions but skewness is not modulated by task difficulty (i.e. ratio). Accordingly, we have to consider that no diffusion process might underlie the data. Furthermore, in 89 out of 384 cases, a Mann-Whitney-U test revealed that RTs significantly differed between correct and error responses. RTs were faster for correct responses in 45 cases (nine or 36, corresponding to children or adolescents, respectively) for the reward and 30 cases (six or 24, corresponding to children or adolescents, respectively) for the control condition (without correction for family-wise error). RTs were slower for correct responses in four cases (one or three, corresponding to children or adolescents, respectively) for the reward and ten cases (six or four, corresponding to children or adolescents, respectively) for the control condition (without correction for family-wise error). The first occasions are indicators of across-trial variability in drift rate, the latter occasions point to across-trial variability in the starting point. Therefore, the EZ-diffusion model probably underestimated all parameters, particularly the drift rate, for these cases ^17^. To test whether the starting point was unbiased ANOVAs with Target (left vs. right) and Response (correct vs. error) on RTs in the different conditions were computed for each participant. Results for two children (reward and control condition) and one adolescent (control condition only) point to a potential prioritization of one answer button (not corrected for family-wise error). Due to this fact and since skewness of the RT distribution was not modulated by task difficulty, the EZ-diffusion model can be applied only with extreme caution.

**References**

1 Dix, A. & Li, S. C. Incentive motivation improves numerosity discrimination: Insights from pupillometry combined with drift-diffusion modelling. *Sci Rep* **10**, 2608, doi:10.1038/s41598-020-59415-3 (2020).

2 Faul, F., Erdfelder, E., Lang, A.-G. & Buchner, A. G*Power 3: A flexible statistical power analysis program for the social, behavioral, and biomedical sciences. *Behavior research methods* **39**, 175-191, doi:10.3758/BF03193146 (2007).

3 Carey, E., Hill, F., Devine, A. & Szűcs, D. The Modified Abbreviated Math Anxiety Scale: A Valid and Reliable Instrument for Use with Children. *Frontiers in psychology* **8**, doi:10.3389/fpsyg.2017.00011 (2017).

4 Carver, C. S. & White, T. L. Behavioral inhibition, behavioral activation, and affective responses to impending reward and punishment: The BIS/BAS scales. *Journal of Personality and Social Psychology* **67**, 319-333, doi:10.1037/0022-3514.67.2.319 (1994).

5 Keller, U. *et al.* A Need for Cognition Scale for Children and Adolescents. *European Journal of Psychological Assessment*, 1-13, doi:10.1027/1015-5759/a000370 (2016).

6 Nosworthy, N., Bugden, S., Archibald, L., Evans, B. & Ansari, D. A two-minute paper-and-pencil test of symbolic and nonsymbolic numerical magnitude processing explains variability in primary school children's arithmetic competence. *PloS one* **8**, e67918, doi:10.1371/journal.pone.0067918 (2013).

7 Woodcock, R. W. & Johnson, M. B. *Woodcock-Johnson Psycho-Educational Battery–Revised*. (Riverside Publishing, 1989).

8 Ekstrom, R. B., French, J. W., Harman, H. & Derman, D. *Kit of factor-referenced cognitive tests (rev. ed.)*. (Educational Testing Service, 1976).

9 Petermann, F. *WISC-IV: Wechsler Intelligence Scale for Children-: Deutschsprachige Adaptation nach D. Wechsler*. (Pearson Assessment and Information, 2011).

10 Lehrl, S. *MWT-B: Mehrfach-Wahl-Wortschatz-Test B*. (Straube, 1977).

11 Nagel, I. E. *et al.* Performance level modulates adult age differences in brain activation during spatial working memory. *Proceedings of the National Academy of Sciences of the United States of America* **106**, 22552-22557, doi:10.1073/pnas.0908238106 (2009).

12 DeWind, N. K., Adams, G. K., Platt, M. L. & Brannon, E. M. Modeling the approximate number system to quantify the contribution of visual stimulus features. *Cognition* **142**, 247-265, doi:10.1016/j.cognition.2015.05.016 (2015).

13 Loewenfeld, I. E. *The pupil: anatomy, physiology, and clinical applications* (Iowa State University Press, 1993).

14 Doughty, M. J. Assessment of short-term variability in human spontaneous blink rate during video observation with or without head / chin support. *Clin Exp Optom* **99**, 135-141, doi:10.1111/cxo.12326 (2016).

15 Hyde, D. C., Khanum, S. & Spelke, E. S. Brief non-symbolic, approximate number practice enhances subsequent exact symbolic arithmetic in children. *Cognition* **131**, 92-107, doi:10.1016/j.cognition.2013.12.007 (2014).

16 Park, J. & Brannon, E. M. Training the approximate number system improves math proficiency. *Psychol Sci* **24**, 2013-2019, doi:10.1177/0956797613482944 (2013).

17 Wagenmakers, E., Van Der Maas, H. L. J. & Grasman, R. P. P. P. An EZ-diffusion model for response time and accuracy. *Psychon Bull Rev* **14**, 3-22, doi:10.3758/BF03194023 (2007).
